# Live Cell Imaging of the Nascent Inactive X Chromosome during the Early Differentiation Process of Naive ES Cells towards Epiblast Stem Cells

**DOI:** 10.1371/journal.pone.0116109

**Published:** 2014-12-29

**Authors:** Aurélia Guyochin, Sylvain Maenner, Erin Tsi-Jia Chu, Asma Hentati, Mikael Attia, Philip Avner, Philippe Clerc

**Affiliations:** 1 Génétique Moléculaire Murine, CNRS URA2578, Institut Pasteur, Paris, France; 2 EMBL Monterotondo, Adriano Buzzati-Traverso Campus, Monterotondo, Italy; 3 Epigénétique des Cellules Souches, Department of Developmental Biology, CNRS URA2578, Institut Pasteur, Paris, France; 4 Université de Technologie de Compiègne, Compiègne, France; 5 Université Blaise Pascal, Clermont-Ferrand, France; Florida State University, United States of America

## Abstract

Random X-chromosome inactivation ensures dosage compensation in mammals through the transcriptional silencing of one of the two X chromosomes present in each female cell. Silencing is initiated in the differentiating epiblast of the mouse female embryos through coating of the nascent inactive X chromosome by the non-coding RNA *Xist*, which subsequently recruits the Polycomb Complex PRC2 leading to histone H3-K27 methylation. Here we examined in mouse ES cells the early steps of the transition from naive ES cells towards epiblast stem cells as a model for inducing X chromosome inactivation *in vitro*. We show that these conditions efficiently induce random XCI. Importantly, in a transient phase of this differentiation pathway, both X chromosomes are coated with *Xist* RNA in up to 15% of the XX cells. In an attempt to determine the dynamics of this process, we designed a strategy aimed at visualizing the nascent inactive X-chromosome in live cells. We generated transgenic female XX ES cells expressing the PRC2 component Ezh2 fused to the fluorescent protein Venus. The fluorescent fusion protein was expressed at sub-physiological levels and located in nuclei of ES cells. Upon differentiation of ES cell towards epiblast stem cell fate, Venus-fluorescent territories appearing in interphase nuclei were identified as nascent inactive X chromosomes by their association with *Xist* RNA. Imaging of Ezh2-Venus for up to 24 hours during the differentiation process showed survival of some cells with two fluorescent domains and a surprising dynamics of the fluorescent territories across cell division and in the course of the differentiation process. Our data reveal a strategy for visualizing the nascent inactive X chromosome and suggests the possibility for a large plasticity of the nascent inactive X chromosome.

## Introduction

Random X chromosome inactivation (XCI) is the mechanism that compensates in mammals for the dosage difference that arises from the different number of X chromosomes in males and females. XCI accomplishes this task by silencing the expression of most genes of a single X chromosome in each cell of the female tissues [1]. The random nature of XCI results in tissues of female mammals being chimeric because each cell will express only X-linked genes of the paternal or the maternal X.

Causal to the transcriptional silencing of the inactive X chromosome is the sequential deposition of several layers of epigenetic regulation during early development of the embryo [2], [3]. The earliest known event, which acts as a trigger for the overall process, is the coating of the nascent inactive X chromosome by the *Xist* non-coding RNA [4]. The parameters of this association have started to be explored in live cells by expressing an MS2-tagged *Xist* RNA from a randomly inserted transgene [5]. A current view is that *Xist* RNA acts as a bait to recruit enzymatic complexes involved in progressively modifying the chromatin structure of the nascent inactive X chromosome. The Polycomb Group Complex 2 (PRC2), which contains the Ezh2 H3K27 methyltransferase, is recruited early, then followed by PRC1 involved in H2A ubiquitinylation [6]. Recruitment of macroH2A and methylation of CpG islands are later features incorporated into the mature inactive X chromosome [2], [3].

In the mouse, random X chromosome inactivation occurs at around day 5.5 postfertilisation in the differentiating epiblast shortly after implantation of the blastocyst and prior to gastrulation [7]. The difficulty in accessing this early embryonic stage has stimulated the interest in stem cells derived from the blastocyst and the study of XCI in cell culture. Female ES cells carry two active X chromosomes and are believed to recapitulate random X inactivation when induced to differentiate. Pluripotent ES cells have been classically maintained in culture in the presence of the cytokine LIF plus Fetal Calf Serum. A more profound state of pluripotency in ES cells cultures has been obtained using serum-free culture conditions supplemented by LIF plus two chemical agents acting on the FGF and β-catenin signaling pathways (2i plus LIF) [8]. Another type of pluripotent cells, corresponding to a « primed » state of pluripotency and known as epiblast stem cells (EpiSCs), has been derived from implanted embryos [9], [10]. Interestingly, female EpiSCs carry one active and one inactive X chromosome [11]. Although protocols have been designed to induce the transition from ES cells to EpiSCs, these experimental conditions have not as yet been exploited for detailed studies of the XCI process. Analyzing the exit from pluripotency along the early transition from ES to EpiSC, a recent report referred to it as generating epiblast-like cells (EpiLCs) [12].

Strict regulatory mechanisms are required to ensure that XCI is controlled by genetic sex and that a single X is randomly chosen in each female cell for inactivation. A mechanism named counting, which senses the X chromosomes number in relation to the autosome number, has been hypothesized to ensure in particular that silencing is not triggered in male cells. However, a complete understanding of counting and random choice currently remains elusive. It is known that the X inactivation center, a regulatory region several hundreds kilobases in size surrounding the *Xist* gene, contributes to choice and counting and controls the onset of *Xist* upregulation [2], [3], [13]. Several elements within the Xic have been identified in targeted mutagenesis approaches. However, mechanistic interpretations of the mutations have been made difficult by our inability to conclude as to the primary or secondary nature of the given phenotype. An absence of cells carrying a particular pattern of XCI can for instance either result from the invalidation of the pathway leading to this pattern or from the mortality of the cells achieving this pattern. Live cell analysis that can address such issues is likely to be determinant to future progress in the field.

Here we have attempted to tackle both the application of advanced differentiation models for XCI studies and live cell imaging of the nascent inactive X chromosome. Analyzing the early transition from ES cells to epiblast-like cells (EpiLCs [12]), we show a normal recapitulation of the counting and choice processes of XCI although with an unusually high frequency of cells in which both X chromosomes are coated by *Xist* RNA. We report on a new strategy to visualize the nascent inactive X chromosome in interphase nuclei of live cells through expression of a transgenic Ezh2-Venus fusion protein. Using this system and time-lapse analysis, we observe a surprising plasticity of the nascent inactive X which is suggestive of new hypotheses for the regulation of the XCI process.

## Materials and Methods

### 2i ES cell culture and derivation

ES cells were routinely cultured in 2i medium [8], [14], [15] supplemented with LIF on gelatin-coated tissue -culture plates in a 5% CO2 humid incubator at 37°C. Passaging every 48 hours was realized after dissociation of cell clumps with accutase (Sigma, France), centrifugation, and re-suspension in culture medium before plating. Derivation of the gC1 ES cell line in 2i medium was performed as previously described from a 129Sv/M. m. castaneus F1 female embryo at 2,5 day post-coitum [14]. All mice were kept at the Institut Pasteur mouse facility. When required, euthanasia was performed by CO2 inhalation and animal sacrifice for embryo collection was done by cervical dislocation. Animal studies for this project have been approved by the relevant institutional review boards (Comité d'éthique en expérimentation animale CEEA 59, IDF, Paris and C2EA 89, Paris) under the protocol number 2013-0043.

### Differentiation of ES cells into EpiLCs

ES cells were routinely cultured in 2i plus LIF medium. After dissociation, extensive washing away of the inhibitors and of the LIF, ES cells were plated on coverslips or in tissue-culture wells coated with laminin (L2020, Sigma) in N2B27 medium depleted for retinoids and supplemented with 25 ng/mL FGF4 and 10 ng/mL Activin A (R&D Systems, Europe). Plating density was 70 000 cells per cm2 for routine experiments. The medium was replaced with fresh medium after 15 hours and one volume of fresh medium was added the second day of differentiation.

### Q RT-PCR

RNA extraction, reverse transcription and Q RT-PCR were performed as previously described [16]. All primers have been described [16] except the primers for *Xist* trans-exonic and non-allelic-assay Xe34 (forward: AGCTTACAGGCCACATGGAG; reverse: CTCCACCTAGGGATCGTCAA).

### RNA-FISH, DNA-FISH, and immuno-fluorescence

RNA, DNA and RNA-DNA-FISH were performed as previously described [16]. DNA from Bac RP24-240J16 was used to generate the *Rnf12* probe. Immuno-fluorescence combined with RNA-FISH was performed as described [17]. Antibodies against H3-K27me3 and Ezh2 were obtained from Millipore (respectively #07-449 and former Upstate #07-689). Fluorescent microscopy and imaging of FISH and immunofluorescence experiments were performed using a Zeiss Axioplan microscope, an Hamamtsu ORCA-AG camera and the Volocity software.

### Protein tagging by BAC recombineering and obtention of the Ezh2-Venus transgenic ES cells

Briefly, using the mouse BAC RP24-359B18 that covers the mouse Ezh2 gene locus, we C-terminally tagged the Ezh2 gene with a 2xTy1-PreS-lox5171-mVenus-lox5171-Biotin-rox-T2A-gb3-Kanamycin-rox-3xFlag cassette in a two-step homologous recombination process in E. coli [18]. DNA from two tagged BACs were electroporated, using Amaxa-nucleofector II (Lonza) into HP3-10 ES cells and selection was performed using Neomycin (Sigma) 350 µg/ml for 9 days.

### Western Blotting

Nuclear extract of ES cells were prepared and analyzed by SDS-PAGE as described [19]. The antibody against Ezh2 (Millipore #07-689) was diluted 2000-fold. Chemiluminescence reagents (ECL, Amersham) and films (Kodak) were used for detection.

### Live cell microscopy and imaging

For live-cell imaging, cells were grown on laminin-coated glass coverslips (Mattek). Wide field microscopy was performed on a Zeiss Observer microscope caged in a 37°C incubator using a 40× oil immersion objective. Cells were maintained at 37°C in a 5% CO2 chamber during the acquisition process. Conditions for time-lapse acquisition were optimized in order to maximize both image quality and cell survival. This led us to acquire Z-stacks of 10 planes with a stepping of 1.2 microns were acquired every 12 minutes for 25 hours. Fluorescence excitation was performed with a 505 nm LED (CooLED) set at 25% of intensity. Images were acquired using an EM-CCD camera (Evolve, Photometrics) set at EM-gain 350 at 10 MHz Gain 3, using an exposure of 400 mSec per plane. One image using transmitted light was also acquired at every time point. Automation of the acquisition and image processing were realized using the software Metamorph.

## Results

### Initiation of X chromosome inactivation during the differentiation of naive ES cells into epiblast-like cells

Gene expression analysis has shown that washing out LIF and the 2i inhibitors and adding FGF plus activin induces differentiation of « naive » ES cells into « primed » EpiSCs [20]. The early phases of this transition was described as generating epiblast-like cells (EpiLCs) [12]. We reasoned that such an experimental setting ideally combines chemical control and minimal perturbation and could constitute a useful system of differentiation for studying *in vitro* the initiation of the XCI process. Several independent female ES cell lines and control male ES cell lines were used in these studies. The gC1 ES cell line has been derived under 2i conditions during the present study and is routinely maintained in 2i plus LIF. The two female ES cell lines PGK1 and HP3-10 and the two male ES cell lines CK35 and the E14T have been initially derived in serum plus LIF [21]–[23] but were readily adapted to 2i plus LIF culture conditions.

**Figure 1:**
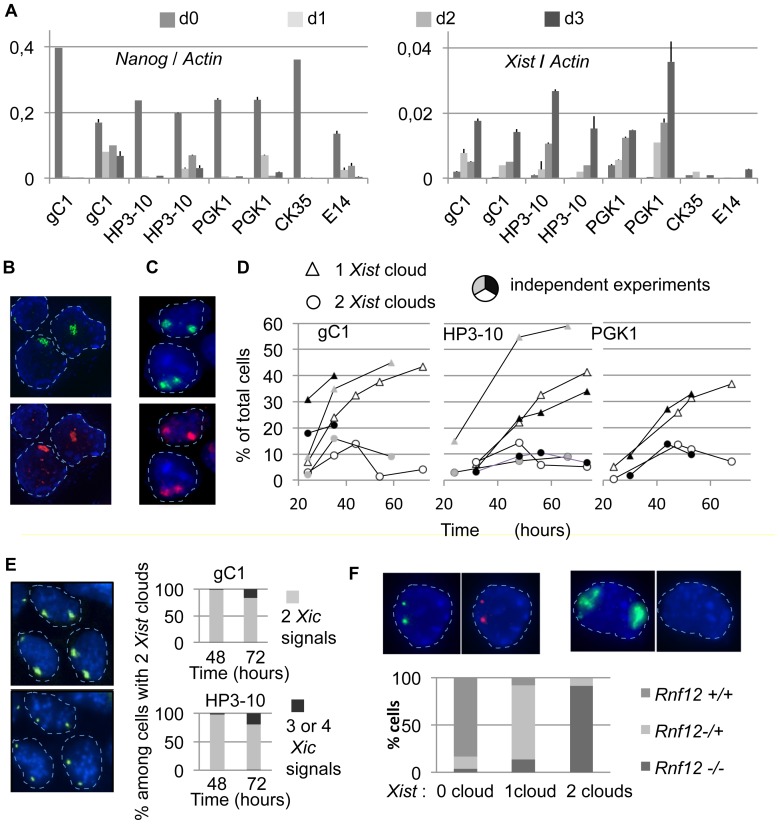
Initiation of X chromosome inactivation can occur on both Xs in the course of differentiation of naive ES cells. A) Kinetic analysis using quantitative RT-PCR of the expression levels of *Nanog* and *Xist* normalized to *Actin* levels in the course of three days of differentiation of ES cells. Wild-type female cell lines: gC1, HP3-10 and PGK1. Control wild-type male cell lines: CK35 and E14Tg2A. RT- controls required at least 10 cycles more than the RT+ samples in order to exceed threshold. Columns represent the ratio of the means of triplicate measurement of 2^-ct^ for *Nanog*, *Xist* and *Actin* at each timepoint in each differentiation experiment. Error bars represent standard deviation of the ratios of *Xist* (or *Nanog*) to *Actin* calculated as: sd of *Xist*/*Actin*  =  (mean of *Xist*/*Actin*) x [(sd of *Xist*/mean of *Xist*)^2^ + (sd of *Actin*/mean of *Actin*)^2^]^0,5^. Two fully independent differentiation experiments performed on separate days with each female ES cell line are shown. B) Immuno-RNA-FISH of *Xist* and H3-K27me3 using the HP3-10 cell line differentiated for 3 days: *Xist* RNA clouds (green, top panel) correspond to domains of enrichment for H3-K27me3 (red, bottom panel). In B, C, E, and F, DAPI blue staining was digitally dampened to favor visualization of the other channels. Nuclei are circled with a dashed line. C) Immuno-RNA-FISH of *Xist* and H3-K27me3 using the gC1 cell line differentiated for 36 hours. Nuclei showing two *Xist* clouds (green, top panel) additionally show two domains of enrichment for the H3-K27me3 mark (red, bottom panel). D) Kinetic analysis using *Xist* RNA-FISH. Cells showing one or two *Xist* clouds were counted over the course of independent 72-hours differentiation experiments with the gC1, HP3-10 and PGK1 ES cell lines (n>250). E) Differentiated female ES cells presenting two *Xist* clouds have a normal complement of two X chromosomes. Sequential RNA-FISH for *Xist* (top left panel) and DNA-FISH using a BAC-561P13 probe which maps within the X inactivation center (bottom left panel) were performed with the HP3-10 cell line. This experiment was similarly performed with the gC1 ES cell line. Cells presenting two Xist clouds were evaluated for their complement of X chromosomes (right panels; gC1 48 h, n = 60; gC1 72 h, n = 25; HP3-10 48 h; n = 45; HP3-10 72 h, n = 30). All or most cells presenting two *Xist* clouds have a normal complement of two X chromosomes, although some X triplody arouse at 72 hours in this cell population. F) Double RNA-FISH for *Xist* (green) and *Rnf12* (red) using the HP3-10 ES cell line differentiated during 40 hours. A cell which did not upregulate *Xist* expressed *Rnf12* bi-allelically (left panels) and a cell which upregulated both alleles of *Xist* silenced *Rnf12* (right panels). The efficiency of detection of the *Rnf12* primary transcription site was 86% as determined in cells lacking *Xist* expression. *Rnf12* expression was examined in cells with no *Xist* cloud (n = 159), with one *Xist* cloud (n = 181) or with 2 *Xist* clouds (n = 75). Nearly all the cells presenting 2 *Xist* clouds were functionally nullisomic for *Rnf12* expression. Repeat experiments with the HP3-10 and gC1 ES cell lines gave essentially identical results.

We controlled for the differentiation of the ES cells and explored *Xist* expression during this process. Quantitative RT-PCR analysis showed a strong reduction of Nanog expression occurring after shifting from 2i plus LIF to EpiLCs culture conditions (Fig. 1A), as previously described [20]. Furthermore, three days after induction we observed a 25- to 100-folds up-regulation of *Xist* expression in female ES cells (Fig. 1A). Under the same conditions, differentiated male ES cells failed to up-regulate *Xist* expression levels (Fig. 1A). It is noticeable that important differences were observed from one experiment to the next (see discussion).

The random choice of a single X chromosome in individual XX cells is an essential characteristic of the XCI process. At the cell population level, the balance of this choice is influenced by a locus termed *Xce* [24]. The female ES cell lines used in this study carry polymorphisms within the *Xist* gene and are heterozygous for *Xce* in a way that would be expected to result in the preferential up-regulation of the 129Sv *Xist* allele. We observed that both *Xist* alleles were up regulated in differentiating ES cells and we found the expected *Xce* effect when analyzing the overall allelic ratio of *Xist* expression (S1 Fig.). This suggests that random choice and *Xce* are functional in differentiating ES cells previously grown in 2i plus LIF conditions.

We next evaluated the formation of nuclear *Xist* RNA clouds and the enrichment of these territories for the histone modification H3-K27me3, two important parameters of the initiation of XCI [2], [3], [13]. Using immuno-RNA-FISH, *Xist* RNA clouds of strong intensity associated with an enrichment in H3-K27me3 were detected as early as 36 hours after initiating differentiation (Fig. 1B). Male cells differentiated in parallel experiments demonstrated a lack of *Xist* clouds (not shown). Although *Xist* RNA clouds were first seen after 24 hours of differentiation, observation of an enrichment in H3-K27me3 at this stage was rare. This may in part be due to the high levels of nuclear staining for H3-K27me3 in early-differentiated cells (not shown).

In conclusion, we have found that essential steps in the process of initiation of XCI were recapitulated during the differentiation of ES cells induced by shifting from 2i plus LIF to EpiLCs culture conditions. Studies of the XCI process *in vitro* could therefore well benefit from this experimental setting.

### *Xist* RNA coating of both X chromosomes is frequent during naive ES to EpiLCs differentiation

Surprisingly, after adapting female ES cells into EpiLCs culture conditions, RNA-FISH experiments showed that many cells presented two *Xist* RNA clouds (Fig. 1C top panel). Since genomic instability is not uncommon in ES cells, a trivial explanation for this could be the presence of three or more X chromosomes in a subset of cells. However, our routine examination of the XX nature of these female ES cell lines would tend to eliminate this explanation. More importantly, sequential RNA-FISH using a *Xist* probe and DNA-FISH using a BAC probe located within the X inactivation center demonstrated that more than 95% of the cells presenting two *Xist* clouds carried only two X chromosomes (Fig. 1E). In these cells, the two *Xist* RNA clouds were large and bright. The *Xist* clouds were associated with an enrichment for H3-K27me3 (Fig. 1C) as well as with the silencing of the *Rnf12* gene (Fig. 1F). In addition, both cells with a single and with two nuclear foci of Ezh2 were detected by standard immunofluorescence during the differentiation process, in agreement with the known association of the PRC2 complex with the nascent inactive X chromosome (S4 Fig.). Clearly, what was occurring in these cells was more than just the temporary burst of *Xist* expression that has been previously described [25].

The kinetics and frequency of bi-allelic upregulation of *Xist* was evaluated by counting the single- and double-cloud cells using RNA-FISH detection. Importantly, both single and double-*Xist*-cloud cells appeared on the same time scale in different cells (Fig. 1D), suggesting that both cell types could be related to each other or at least might be responding to concomitant signaling cues. However, the outcome of both cell types was radically different, with single-*Xist*-cloud cells increasing in number and double-*Xist*-cloud cells progressively disappearing from the cultures after day 2 of differentiation (Fig. 1D). To attempt to address the cell lineage and mechanistic issues associated with these observations, we next designed and exploited a system for visualizing and tracking the nascent inactive X chromosome territories in living cells.

### Production of transgenic female ES cells expressing an Ezh2-Venus fusion protein

**Figure 2:**
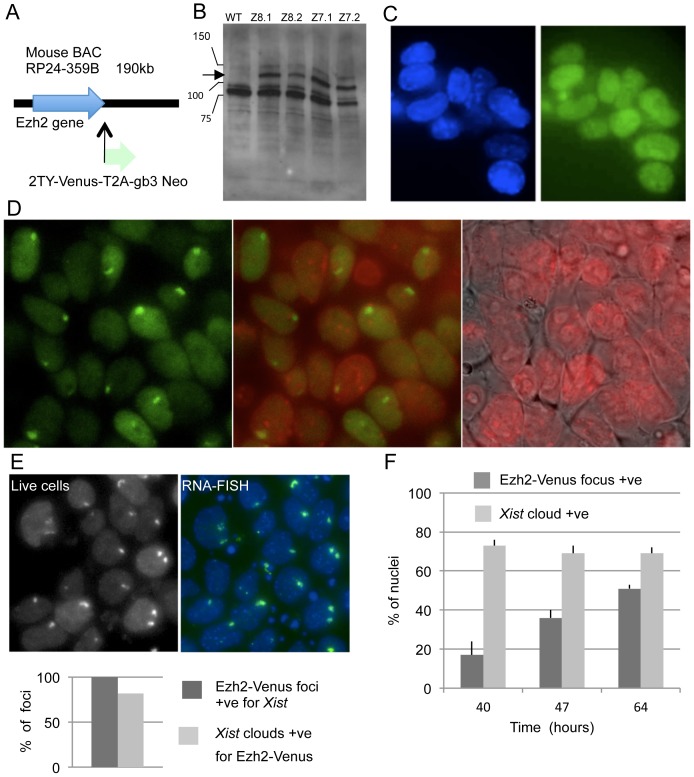
The Ezh2-Venus protein is recruited at the nascent inactive X chromosome in differentiated ES cells. A) Schematic strategy for the COOH-tagging of the Ezh2 protein expressed from a mouse BAC DNA. B) Expression of the Ezh2-Venus fusion protein in ES cells. Western blotting using an Ezh2 antibody and nuclear extracts from the parental ES cell line HP3-10 (WT) and from four neomycin-resistant clones transfected with Venus-tagged BAC DNA The arrow points to the migration level of the fusion protein. C) Nuclear localization of the Ezh2-Venus fusion protein in fixed ES cells. Wide-field fluorescent microscopy for Hoechst 33342 (blue, left panel) and Venus (green, right panel) of the Z8.1 ES cells cultured in 2i plus LIF and fixed for 3 minutes with 4% PFA. D) Ezh2-Venus nuclear foci are detectable in differentiated live female ES cells. Live imaging was performed on the Z8.1 ES cells after differentiation for 50 hours. Nuclear fluorescent foci of Venus signal are visible (green, left and central panel). DNA was stained with Hoechst 33342 (pseudo-colored in red) and overlaid on the phase contrast image (right panel) and on the Venus channel image (central panel). E) Ezh2-Venus nuclear foci correspond to *Xist* RNA clouds. Live cells of the Z8.1 line differentiated during 70 hours were imaged for Venus (top-left panel) and then fixed and processed for *Xist* RNA-FISH (top-right panel; DAPI blue, *Xist* green). Despite moderate shifts due to live cell movements prior to fixation, both panels show recognizable nuclei presenting similarly localized Ezh2-Venus and *Xist* nuclear territories. All nuclear Ezh2-Venus foci detected in live cells corresponded to a *Xist* RNA cloud (bottom panel, n = 60) although the reciprocal was not the same. F) In the course of differentiation, the kinetics of Ezh2-Venus foci is delayed as compared to the kinetics of *Xist* RNA accumulation. Duplicate samples at different time-points of the same differentiation experiment using the Z8.1 ES cell line, were processed for live imaging of Ezh2-Venus or for *Xist* RNA-FISH. Cells were counted for nuclear fluorescent territories after image acquisition. Bars on top of the columns represent standard deviation of the counts of three groups of cells (n>150 each) at each timepoint. Detection of Ezh2-Venus territories is delayed as compared with *Xist* clouds, which had already reached a plateau by 40 hours of differentiation in this experiment. The data presented in panel C to F have been reproduced with no significant difference for at least two other transgenic Ezh2-Venus ES cell lines.

Tagging *Xist* RNA or tagging protein components of the Polycomb repressive complex 2 (PRC2) constitute possible candidate approaches allowing visualization of nascent inactive X chromosome territories in live cells. The more straightforward approach of protein-tagging was more successful in our hands. An Ezh2 BAC clone containing large genomic regions 5′ and 3′ of the gene was used in order to maximize the chances that it contained the necessary regulatory sequences. Using a BAC recombineering strategy [18], the open-reading frame of the fluorescent protein Venus was fused with the COOH-terminal part of Ezh2 (Fig. 2A). Following transfection of the HP3-10 ES cell line and selection using neomycin, transgenic clones were expanded and analyzed by Western blotting using an anti-Ezh2 polyclonal serum. As compared with the parental cell line, recombinant clones showed an additional band whose migration fits with the theoretical molecular weight of the recombinant protein (Fig. 2B). The moderate level of expression of the recombinant protein, from one-half to one-third that of the endogenous Ezh2, would likely not perturb cell physiology. In addition, the fixed ES cells showed a homogeneous fluorescence of the nucleoplasm using wide field microscopy and appropriate filter sets (Fig. 2C), in agreement with the known properties of Ezh2. The weakness of the fluorescent signal, at the limit of visibility by eye, likely resulted from both the low levels of expression of Ezh2-Venus construct and signal reduction caused by formaldehyde fixation. Analysis of 14 different clones transfected with two different recombinant BAC clones did not identify ES cells clones with markedly higher fluorescent intensity.

### Visualizing nascent inactive X nuclear territories in living cells

We next attempted to visualize the nascent inactive X chromosome territories in living cells using wide-field microscopy. In living ES cells differentiated for 3 days, fluorescent nuclear territories could be detected (Fig. 2D) in a subset of cells. Identification of these Ezh2-Venus territories as nascent inactive X chromosomes was achieved using a *Xist* RNA-FISH probe. Field coordinates were recorded while imaging the living cells, followed by cell fixation, RNA-FISH labeling and repositioning of the samples to image the same fields. In all cases, nuclei showing Ezh2-Venus foci also showed *Xist* RNA territories of similar number and morphology (Fig. 2E). Kinetic analysis of the Ezh2-Venus and *Xist* territories in parallel samples in the same differentiation experiment showed that detection of Ezh2-Venus territories was delayed as compared to that of the *Xist* RNA territories (Fig. 2F). This is concordant with Ezh2 being recruited by *Xist* RNA [26] and likely explains occasional differences in intensity observed between both types of territories (Fig. 2E).

We conclude that the Ezh2-Venus fusion protein faithfully labels the nascent inactive X chromosome territory in living cells during the ES cell differentiation process.

### Live cell dynamics of the nascent inactive X chromosome territories

**Figure 3:**
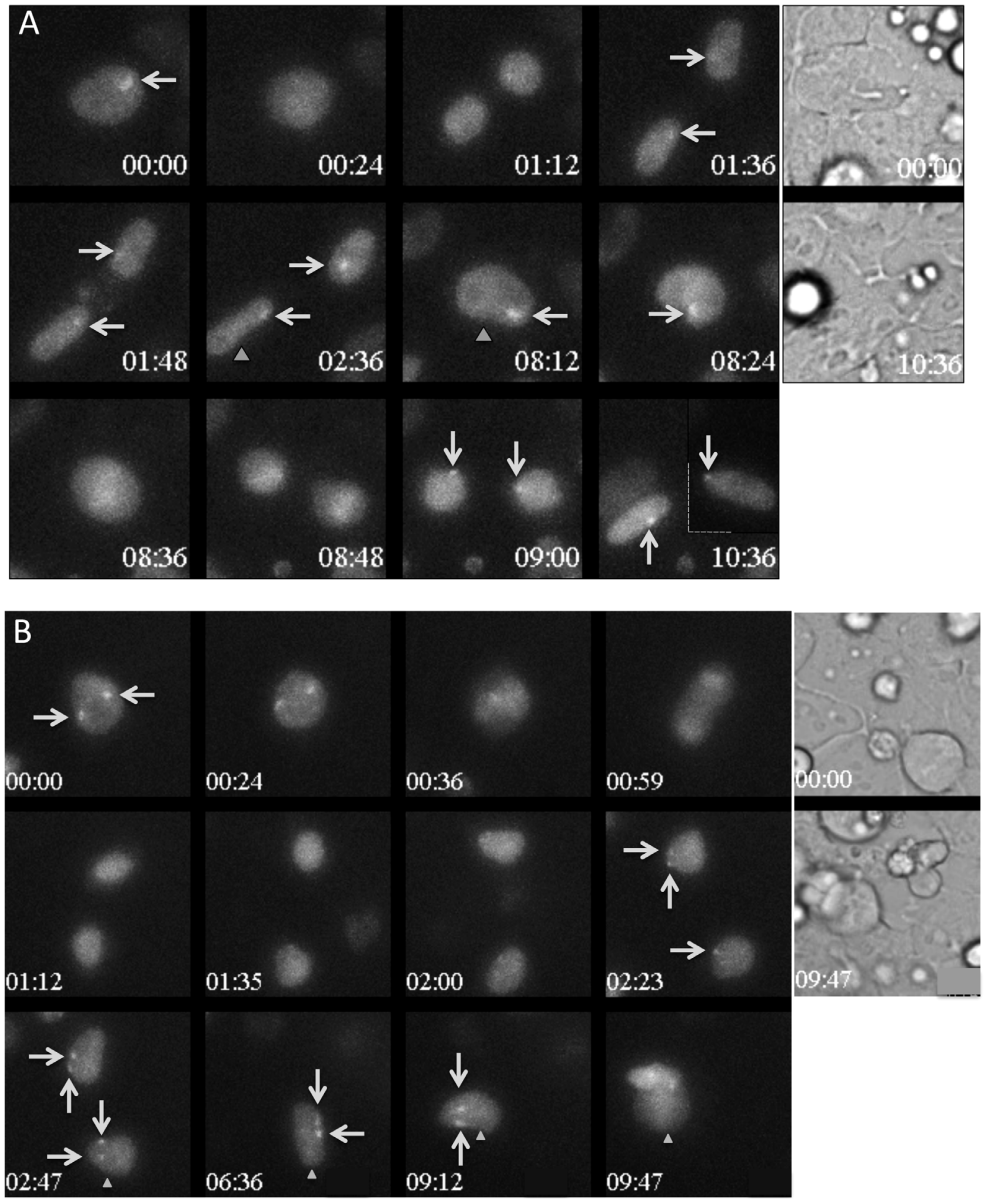
Live-cell imaging showing that the recruitment of Ezh2-Venus to the nascent inactive X chromosome is lost during mitosis and is restored progressively during interphase. In both A and B, the 12 left panels show wide-field fluorescent maximum projection images of Z-stacks in the Venus channel at selected time points during differentiation of the Z8.1 ES cell line. Time is indicated relative to the start of the sequence shown (hours: minutes). The white arrows point to the Ezh2-Venus accumulation foci. When necessary, gray triangles unambiguously identify the cell, which can be followed in two successive panels. Transmission images at the start and the end of the sequence are shown in the two panels on the right. It was verified that the nuclei had been fully imaged in Z by examining individual plans of the Z-stacks. Full time-lapses corresponding to these stills are shown in S2 Video and S3 Video. A) The initial time in this sequence corresponds to 50 hours and 50 minutes after shifting from 2i plus LIF to EpiLCs culture conditions. Daughter nuclei lacked fluorescent Ezh2-Venus accumulation for 1 hour after the first mitosis, and for only 12 minutes after the second mitosis. The dashed line at time 10:36 indicates that the image of one of the cells was digitally treated to bring it closer to its sister. B) The initial time shown in this sequence corresponds to 53 hours and 25 minutes after shifting from 2i plus LIF to EpiLCs culture conditions. After the separation of the daughter cells, the nuclei lacked a domain of Ezh2-Venus accumulation for 1 hour and 12 minutes. The daughter cell in the lower region of the image then started to display a single fluorescent domain and only then regained a second domain (arrows).

**Figure 4:**
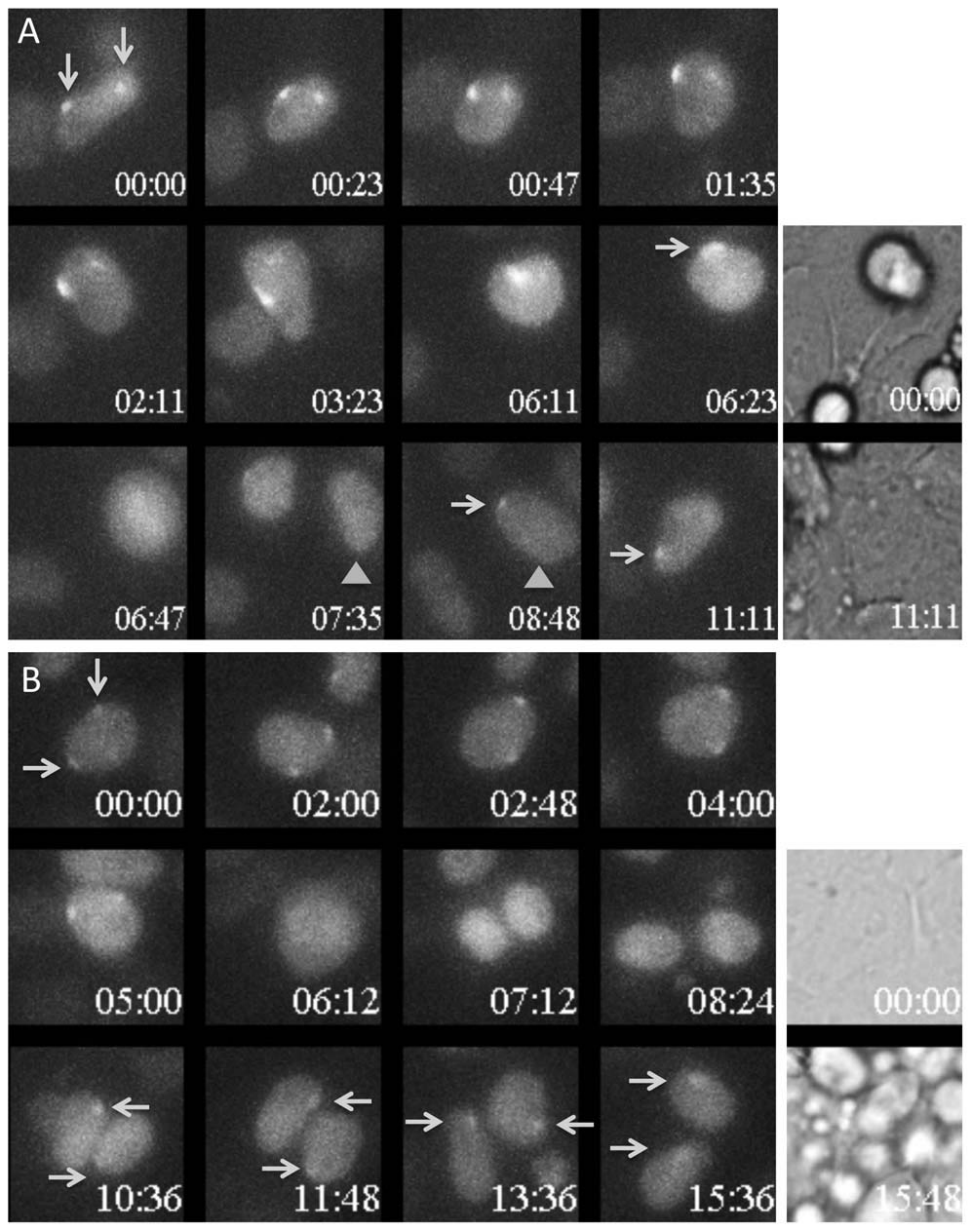
Cells presenting two nuclear domains of accumulation of Ezh2-Venus demonstrate occasional instability in Ezh2-Venus recruitment. In both A and B, the 12 left panels show wide-field fluorescent maximum projection images of Z-stacks in Venus channel at selected time points during differentiation of the Z8.1 ES cell line. Time is indicated relative to the start of the sequence shown (hours: minutes). The white arrows point to the Ezh2-Venus accumulation foci. When necessary, gray triangles allow unambiguous identification of the cell that could be followed in two successive panels. Transmission images at the start and the end of the sequence are shown in the two panels on the right. It was verified that the nuclei had been fully imaged in Z by examining individual plans of the Z-stacks. Full time-lapses corresponding to these stills are shown in S4 Video and S5 Video. A) The initial time in this sequence corresponds to 44 hours and 50 minutes after shifting from 2i plus LIF to EpiLCs culture conditions. In this cell with two fluorescent territories (arrowed), one of the two Ezh2-Venus territories of accumulation faded away with a 6 hours time course. 3 hours and 30 minutes after mitosis, one of the daughter cells still presented a single fluorescent territory (arrowed). B) The initial time in this sequence corresponds to 42 hours and 35 minutes after shifting from 2i plus LIF to EpiLCs culture conditions. Daughter cells exhibited only a single Ezh2-Venus territory of accumulation over more than 6 hours despite the fact that the mother cell had two domains (arrows).

Our initial goal in performing live cell experiments was to determine the fate of cells presenting two nascent inactive X-chromosomes. However, performing long-term time-lapse wide-field fluorescent microscopy using the Ezh2-Venus differentiated ES cells has proved challenging due to a combination of unfavorable features associated with our biological system. This required three types of careful adjustments. Firstly, ES cells differentiating along this pathway demonstrated an accelerated cell cycle (8,75±1,25 hours as measured by live-cell imaging using phase-contrast microscopy, n = 26). However cell number only doubled per day due to a high frequency of concomitant cell death. This resulted in a layer of dead cells on top of the live cells (an example is shown in the late transmission view in Figs. 3B and 4B). To preserve fluorescent imaging quality, these dead cells were therefore flushed away from time to time. Secondly, efficient tracking of single cells for several hours required a large space between the fluorescent cells and therefore low plating densities. Differentiating cells are however highly sensitive to low cell density which resulted in an increased cell mortality and a number of experimental failures. We resolved these opposing constraints by mixing, prior to the start of the differentiation experiment, transgenic Ezh2-Venus ES cells with supportive wild-type non-fluorescent parental ES cells in a ratio of 1 to 3. Thirdly, the fluorescent signal of Ezh2-Venus was weak and the differentiating ES cells demonstrated significant photosensitivity. Optimization of several parameters and compromise on the issue of image quality have allowed this issue to be circumscribed. This has included use of an EM-CCD camera associated with a low intensity of excitation light, the use of a low magnification lens (40×) and acquisition of Z-stacks made up of a limited number of planes with a large stepping (10 plans with 1.2 microns steps). Finally we have spaced out time-lapse acquisitions to the maximum interval still compatible with satisfying cell tracking (12 min.). These settings allowed us to record living cells in our system for up to 24 hours.

A likely destiny of the cells with two nascent inactive Xs is more or less rapid death due to functional nullisomy for X-linked genes. However, the intense cell mortality occurring during the differentiation process precluded us from identifying significant differences in cell mortality correlated with the number of observed fluorescent territories in the cells. Nevertheless, some estimate of the survival capacity of the cells could be made using live imaging. In the course of four successful recordings lasting 24 hours and constituted each of 40 acquired fields, we managed to track across two successive mitosis, eight individual cells presenting a single Ezh2-Venus-enriched territory and some of their descendants. In the same data set, three cells presenting two fluorescent territories could be followed over the same duration. The time-lapse recording of one such cell is shown in the S1 Video. The three cases that we observed show that cells with two nascent inactive X chromosomes can undergo two successive mitosis and survive for at least 24 hours. In agreement with an unchanged fitness, the length of the cycle of these cells was not found to be significantly different from the length of the cell cycle in the total cell population. No conclusions can however be drawn concerning the vitality of the overall population of cells with two nascent inactive Xs.

We have examined the stability of the Venus fluorescent territories in relation to cell division (stills in Fig. 3A and B and the corresponding S2 Video and S3 Video). In both cells with a single and two nascent inactive Xs, fluorescent territories were maintained up to shortly before the cells rounded up for mitosis. Partial loss of focus of the rounded cells might explain why a fluorescent chromosome was not detected in mitotic cells. However cells previously presenting one or two fluorescent territories before mitosis were nearly always devoid of Ezh2-Venus foci when flattening and re-entering interphase. This strongly suggests that, in these cells, Ezh2-Venus was released from the nascent inactive X chromosome at some point during mitosis. The average delay for reappearance of the fluorescent territories after mitosis was not significantly different in cells with a single or with two fluorescent territories (1,5+−0.5 hour, n = 24 and 12 respectively). In both cell types, fluorescent territories often gained in intensity during progression of the cell cycle (Fig. 3A and 3B). An additional level of complexity, probably reflecting the dynamics of differentiation, is that the delay of recruitment of Ezh2-Venus after cell division was frequently longer at early timepoints than at later timepoints of differentiation. A cell that was tracked for two successive mitosis showed a longer delay of recruitment after the first mitosis than after the second mitosis (Fig. 3A).

We next addressed the persistence of the fluorescent territories in cells with two nascent inactive X chromosomes. We observed in three instances the fading away of one of the two territories of an interphase nucleus, a fading that occurred progressively over several hours (Fig. 4A). Significantly, at least one in each pair of daughters of these cells showed a single territory for several hours, which may suggest that this converted state was stable. We observed more frequently cells with two fluorescent territories that produced after division two daughter cells each with a single fluorescent territory (Fig. 4B). This two-to-one reverted situation was maintained for 6 hours in the example shown (Fig. 4B) It is however unclear whether this situation is then stable. In several cases we observed that the re-appearance of the two territories post-mitosis was not perfectly synchronous, occurring with a delay of one or two frames between the two territories (Fig. 3B). An extreme asynchrony was observed on three occasions in which the recruitment of Ezh2-Venus on one X chromosome preceded the recruitment on the other by more than 2.5 hours (S2 Fig.). Asynchrony in the recruitment of Ezh2-Venus on the two X chromosomes may surprisingly suggest that the two chromosomes are not equivalent in these cells. At this stage, it cannot be excluded that other cells presenting two fluorescent territories prior to cell division and a single one after cell division, might not constitute temporary situations since these cells could not be tracked up to the next mitosis.

In conclusion, we have found that the recruitment of Ezh2-Venus presents surprising levels of heterogeneity and stochasticity among individual cells, and is influenced by differentiation progression, cell cycle and even chromosomal individualization. These properties preclude us at the moment from reaching a definitive conclusion concerning the cell lineage relationship between the cells which presented a single- and two nascent inactive X chromosomes.

## Discussion

We have analyzed in detail a novel ES cell differentiation protocol directing cells from serum-free 2i plus LIF conditions towards epiblast-like cells, for its capacity to induce the initiation of X chromosome inactivation. In addition to efficiently recapitulating essential features of X chromosome inactivation, this differentiation protocol resulted in significant levels of XX cells initiating inactivation on both X chromosomes within a single cell, a state referred to in the present section as dualXi cells. In an attempt to document the fate of these cells, we designed a system that enabled us to visualize the nascent inactive X chromosome in living cells by expressing an Ezh2-Venus fusion protein in female differentiated ES cells. Time-lapse analysis of differentiating cells over 24 hours allowed us to detect dualXi individual cells surviving and proliferating for this entire duration. Once established, the recruitment of Ezh2-Venus on the nascent inactive X chromosomes of these cells showed different forms of plasticity which may result from unexplored layers of complexity of the X inactivation process.

We had expected that differentiation of naive ES cells into EpiLCs would have provided us with a high performance setting for live cell imaging of the random XCI process. Whilst our experimental conditions recapitulated random XCI at least as efficiently and accurately as other previously described systems, they did not provide the reproducibility which we had expected from chemically controlled conditions. In particular the percentage of cells failing to upregulate *Xist* expression not only remained significant but in addition varied from one experiment to the next. Although it may be argued that 2i plus LIF results in a more homogeneous expression level of NANOG protein in the cell population, heterogeneity linked to the cell cycle and/or the size of the cell colony will likely also influence the response to differentiation cues. Indeed we believe the links existing between signaling, cell cycle and lineage specification to probably be at the root of the residual variability that we have observed. Another feature of this differentiation system is the concomitant high cell mortality that considerably handicapped the implementation of our experimental approaches. The differentiation scheme we used supposedly recapitulates transition between closely related embryological stages, namely the pre-implantation to the post-implantation epiblast stages. We believe the high cell mortality we have observed is likely due to incompleteness of the signaling cues used *in vitro*, although we cannot completely ignore the possibility that the *in vivo* process itself is also widely accompanied by cell death.

The frequent occurrence of dualXi cells in XX cells, the respect of the *Xce* effect and the functional sensing of the X chromosome number that resulted in a lack of initiation in male cells, are highly significant features of the differentiation process that we have implemented. Whilst diploid female ES cells differentiated into embryoid bodies have previously been reported to form two *Xist* Clouds [27] the frequency of such events did not exceed 3% of the total cell population in this report [27]. Furthermore, the ES cell lines gC1, PGK1 and HP3-10 cultured in serum plus LIF and differentiated by LIF removal and low cell density culture showed a very limited number of dualXi cells (less than 2 percent at any timepoint; S3 Fig.). This is very different from the 10–15% of dualXi cells we observed using these same three independent female ES cell lines. We surmise that the pluripotency network and/or specificity of the dynamics of the differentiation process used here, are responsible for the propensity to initiate frequently XCI bi-allelically. This conclusion is supported by RNA interference experiments targeting Oct4 in embryoid bodies which resulted in a low frequency of cells which up-regulated *Xist* bi-allelically [28].

The bi-allelic XCI we have observed can lead to the accumulation of H3-K27me3 and silencing of gene expression as demonstrated by RNA-FISH analysis of *Rnf12*. The bi-allelic initiation of XCI we have observed therefore seems more advanced than that associated with the transient bi-allelic up-regulation of *Xist* which has been reported for retinoic differentiated female ES cells [25] and suggested to represent a normal step on the way toward random XCI. If bi-allelic upregulation was part of the normal process of XCI, an early mechanism responsible for the transition from bi- to mono-allelic *Xist* expression might fail more or less frequently, depending on the signaling and kinetics of the differentiation, leading to the phenotype we have observed. The finding of widespread bi-allelic *Xist* up-regulation in early human and rabbit embryos [29] may reflect species dependent differences in early embryonic differentiation/signaling pathways. In some mammals, a smaller size of female relatively to male embryos has been observed in the early post-implantation phase [30], [31]. This could be related to a higher cell mortality, possibly indicative of initiation of X inactivation on both Xs in a subset of cells, although alternative explanations cannot be eliminated.

We successfully used an Ezh2-Venus fusion protein to visualize the nascent inactive X chromosome in living cells. A MacroH2A-GFP fluorescent fusion protein was previously used to visualize the inactive X chromosome in living cells of the embryo and of the adult [32]. One may argue from a theoretical perspective that using an early marker of the inactive X chromosome may be preferable to a late marker in order to visualize the initiation of XCI. Several properties of Ezh2 appeared appropriately conserved in the modified Ezh2-Venus protein. These include the propensity for recruitment of Ezh2-Venus at X chromosome territories at high frequency and only following *Xist* up-regulation and accumulation. In addition and similarly to the endogenous Ezh2 protein [33], the association of Ezh2-Venus with the nascent inactive X chromosome was transient and became undetectable after 7 days of differentiation (data not shown). The kinetics of signal loss during mitosis and the delay of recruitment of Ezh2-Venus to the nascent inactive X following cell division in early differentiation stage seem however to differ from that reported for Ezh2 on the mitotic inactive X chromosome in both TS cells [34] and differentiating ES cells [35] using immunofluorescence analysis. Further studies will address statistically the association of Ezh2 with the inactive X during mitosis depending on the cell lineage and differentiation pathway.

Whilst our results have not achieved statistical significance they clearly show examples of dualXi cells with vitality maintained for in excess of 24 hours. The supposed stringency and speed of counter-selection for inappropriate X inactivation configurations has been a critical element in interpreting whether a given X inactivation phenotype results from primary mechanisms or secondary effects related to cell death. Therefore our observations should be kept in mind for future interpretation of the phenotypes of mutations targeted to within the X inactivation center. Our observations moreover do not support a previously formulated version of *Rnf12* control of *Xist* expression based on a probabilistic allele autonomous X inactivation model [27]. Indeed, we showed bi-allelic transcriptional silencing of *Rnf12* occurring in the dualXi cells, suggesting that *Rnf12* concentration drops did not rapidly induce a repression of *Xist* levels. This seems to fit a recent report showing that *Rnf12* is dispensable for random XCI in mice [36].

Fluctuations in the association of Ezh2-Venus with nascent inactive X chromosome territories may suggest the possibility that nascent inactive X chromosomes are sufficiently plastic for reversion to the active state to occur. Such a hypothesis has previously been formulated in regard of two biological contexts [37], [38]. At this stage however it is uncertain whether our observations result from a reversion of several features associated with the nascent inactive X chromosomes, to the plasticity of the Ezh2 Xi association, or to an alteration in Ezh2 anchorage caused by its Venus fusion partner. Whilst such an alteration is possible it would be surprising given the otherwise normal association of the Ezh2-Venus protein with the nascent inactive X chromosome. Irrespective of this the prolonged asymmetry of the Ezh2-Venus association that we have observed in some dualXi cells intriguingly suggests that the two nascent inactive X chromosomes are not equivalent.

The present report is among the first of its kind allowing visualization of the nascent inactive X chromosome in live cells. We strongly believe that such dynamic approaches will provide important mechanistic clues about the X inactivation process and that our live-imaging system will contribute to this approach.

## Supporting Information

S1 Fig **The Xce effect on** ***Xist*** **allelic upregulation is detectable in female ES cells differentiated by shifting from 2i plus LIF to EpiSC culture conditions.** Allelic Q RT-PCR measurements of *Xist* RNA in the PGK1 and HP3-10 ES cell lines over a three days time course. Crude values of 2^-CT^ are plotted for spliced *Xist* RNA expressed from the 129Sv allele and from the PGK- 1a alleles. Allelic specificity and amplification efficiencies of the primer pairs were verified in preliminary experiments. The mean allelic % (*Xist*-129Sv)/*Xist* total) considering all the time point is 63% for the PGK1 cell line and 71% for the HP3-10 cell line. Both values are in agreement with published observation in an Xce a/Xce c genomic background.
(TIF)

S2 Fig **An example of asynchronous recruitment of Ezh2-Venus on X chromosomes following cell division.** Wide-field fluorescent maximum projection images of Ezh2-Venus at selected time points during differentiation of the Z8.1 ES cell line. Time is indicated relatively to the start of the sequence shown (hours: minutes). Transmission images at the start and the end of the sequence are shown in the two last panels on the right. It was verified for nuclei lacking an Ezh2-Venus territory that these nuclei had been fully imaged in Z. The dashed line at time 6:47 signals that the image of one of the cell was digitally brought closer to its sister cell. A full time-lapse corresponding to these stills is shown in S6 Video. The cell indicated by a gray triangle shows a single fluorescent territory during almost 3 hours before gaining a second fluorescent territory.
(TIF)

S3 Fig **ES cells differentiated under classical differentiation methods show very limited number of cells with two** ***Xist*** **clouds.** The ES cell lines gC1, HP3-10 and PGK1 were cultured for three passages or more in serum plus LIF medium and differentiated by LIF removal and low cell density culture. A kinetic analysis using *Xist* RNA-FISH was performed in which cells showing one or two *Xist* clouds were counted over the course of 72-hours differentiation experiments with the three ES cell lines. Bars represent standard deviation of the counts of three groups of cells (n>250 each) for each cell line at each timepoint.
(TIF)

S4 Fig **Immunofluorescence detection of Ezh2 using HP3-10 ES cells differentiated for 50 hours under the epiLCs protocol.** Bottom panel: DAPI staining. Middle panel: Ezh2 signal alone. Top panel: Ezh2 and DAPI surimposed. Cells with a single nuclear foci of Ezh2 as well as cells with two nuclear foci of Ezh2 are present.
(TIF)

S1 Video **A cell showing 2 Ezh2-Venus fluorescent nuclear territories performed 2 cell divisions over a 24-hours tracking.** Cells of interest are pointed at certain times with an arrow.
(MP4)

S2 Video **Corresponding to** **Fig. 3A** **.**
(MP4)

S3 Video **Corresponding to** **Fig. 3B** **.**
(MP4)

S4 Video **Corresponding to** **Fig. 4A** **.**
(MP4)

S5 Video **Corresponding to** **Fig. 4B** **.**
(MP4)

S6 Video **Corresponding to** **S2 Fig** **.**
(MP4)
