# Supplementary figures and images for: Live Cell Imaging of the Nascent Inactive X Chromosome during the Early Differentiation Process of Naive ES Cells towards Epiblast Stem Cells
Source: PLoS One. 2014 Dec 29;9(12):e116109. doi: 10.1371/journal.pone.0116109 (PMC4278889; doi:10.1371/journal.pone.0116109)

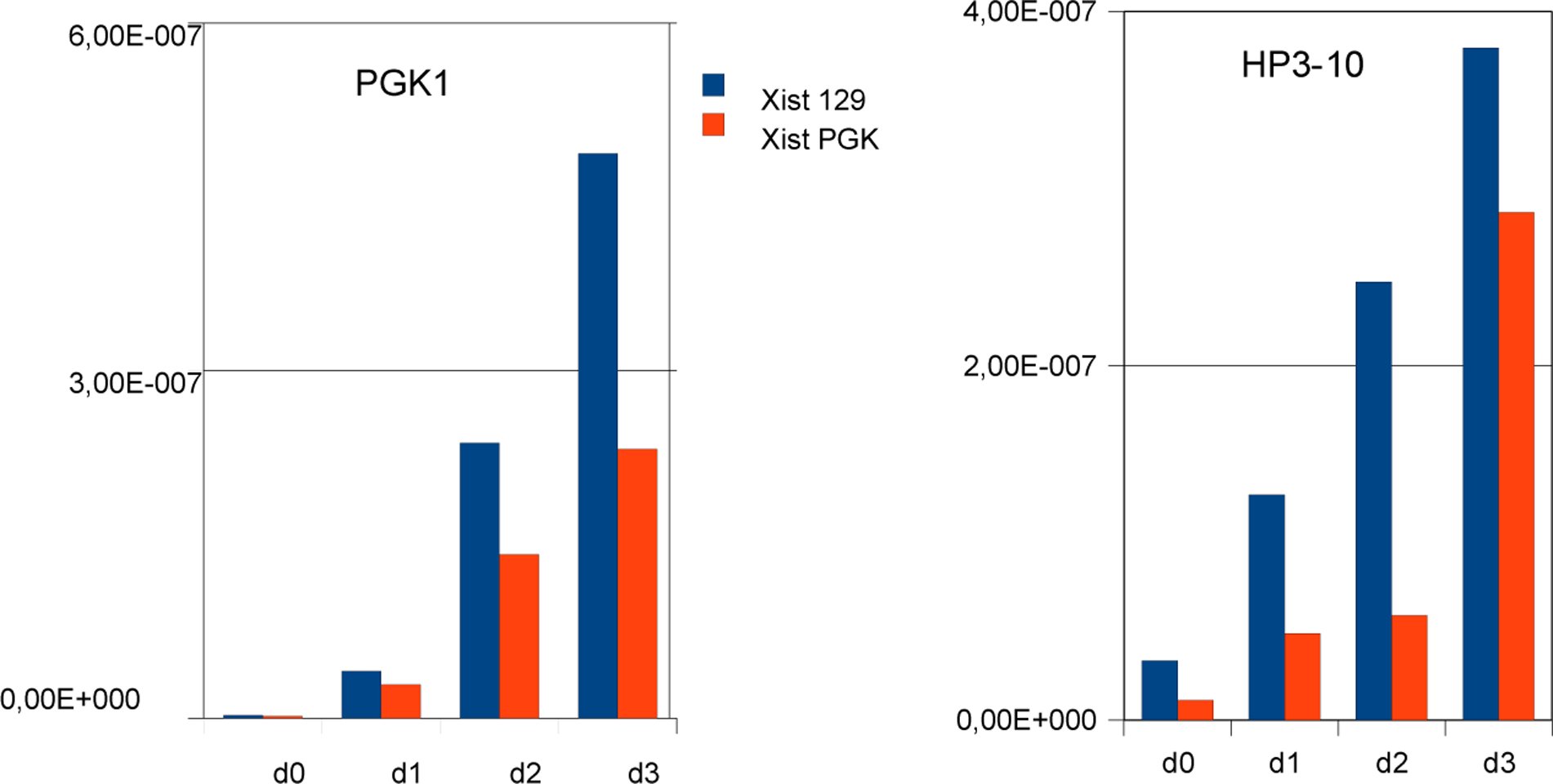

Supplement: S1 Fig — The Xce effect on Xist allelic upregulation is detectable in female ES cells differentiated by shifting from 2i plus LIF to EpiSC culture conditions. Allelic Q RT-PCR measurements of Xist RNA in the PGK1 and HP3-10 ES cell lines over a three days time course. Crude values of 2-CT are plotted for spliced Xist RNA expressed from the 129Sv allele and from the PGK- 1a alleles. Allelic specificity and amplification efficiencies of the primer pairs were verified in preliminary experiments. The mean allelic % (Xist-129Sv)/Xist total) considering all the time point is 63% for the PGK1 cell line and 71% for the HP3-10 cell line. Both values are in agreement with published observation in an Xce a/Xce c genomic background. (TIF) [file pone.0116109.s001.tif]

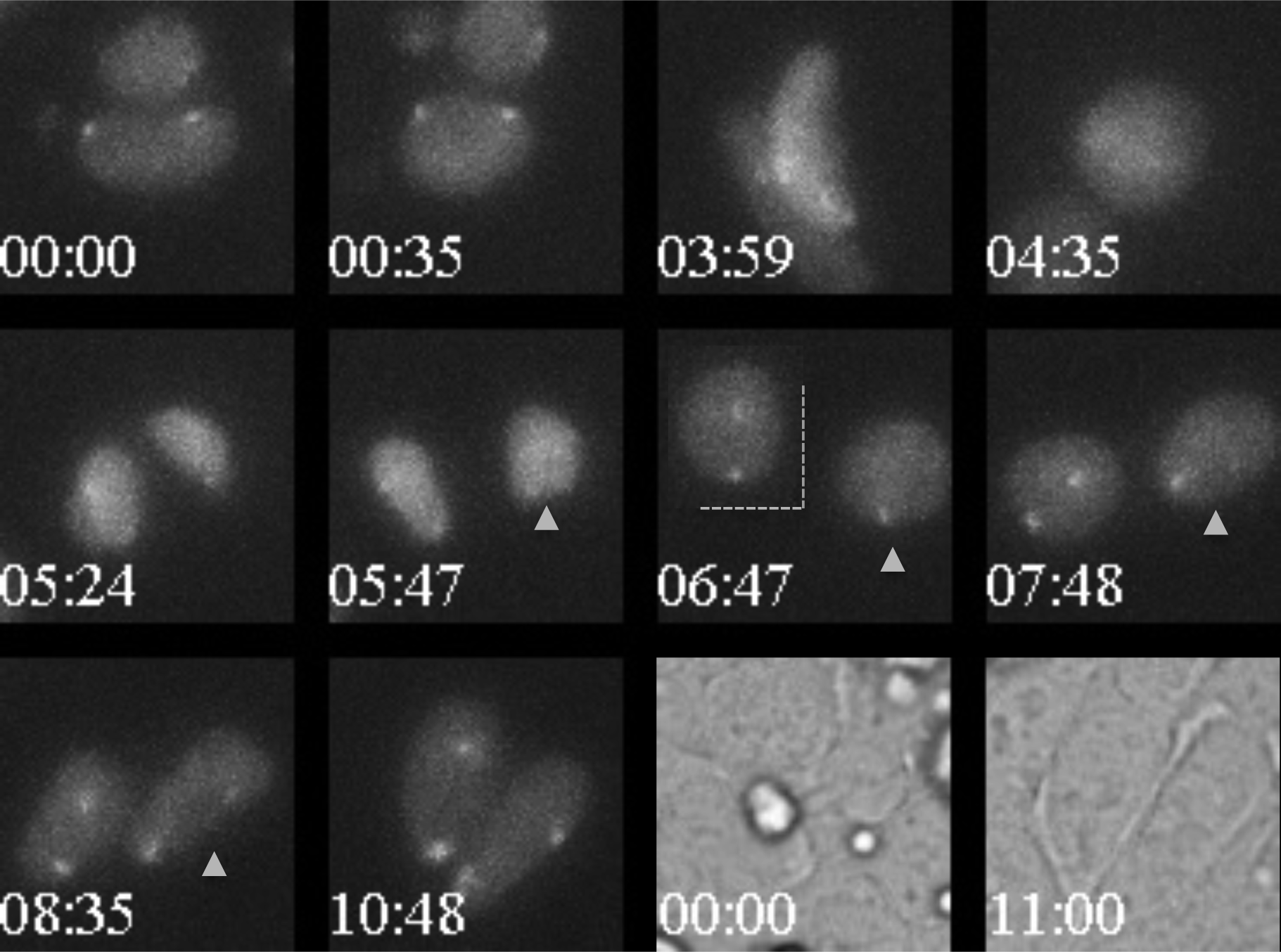

Supplement: S2 Fig — An example of asynchronous recruitment of Ezh2-Venus on X chromosomes following cell division. Wide-field fluorescent maximum projection images of Ezh2-Venus at selected time points during differentiation of the Z8.1 ES cell line. Time is indicated relatively to the start of the sequence shown (hours: minutes). Transmission images at the start and the end of the sequence are shown in the two last panels on the right. It was verified for nuclei lacking an Ezh2-Venus territory that these nuclei had been fully imaged in Z. The dashed line at time 6:47 signals that the image of one of the cell was digitally brought closer to its sister cell. A full time-lapse corresponding to these stills is shown in S6 Video. The cell indicated by a gray triangle shows a single fluorescent territory during almost 3 hours before gaining a second fluorescent territory. (TIF) [file pone.0116109.s002.tif]

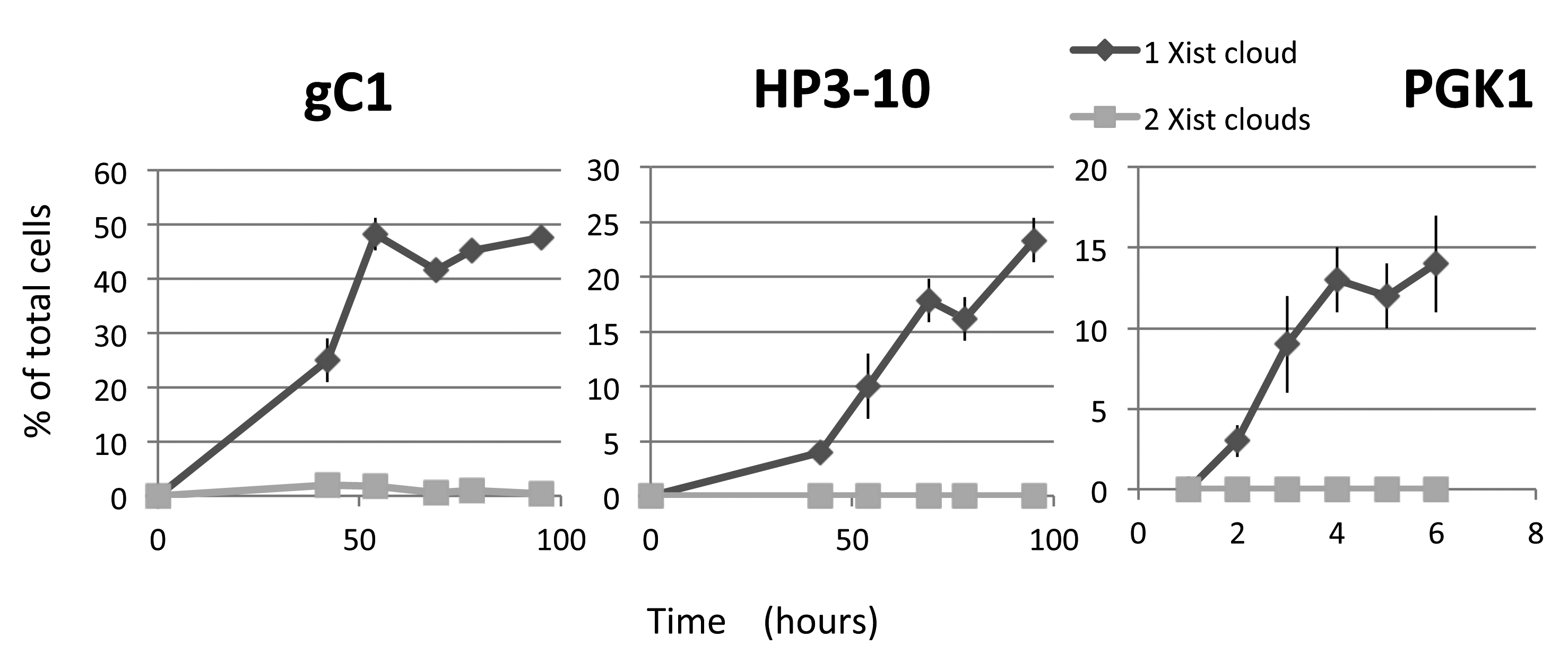

Supplement: S3 Fig — ES cells differentiated under classical differentiation methods show very limited number of cells with two Xist clouds. The ES cell lines gC1, HP3-10 and PGK1 were cultured for three passages or more in serum plus LIF medium and differentiated by LIF removal and low cell density culture. A kinetic analysis using Xist RNA-FISH was performed in which cells showing one or two Xist clouds were counted over the course of 72-hours differentiation experiments with the three ES cell lines. Bars represent standard deviation of the counts of three groups of cells (n>250 each) for each cell line at each timepoint. (TIF) [file pone.0116109.s003.tif]

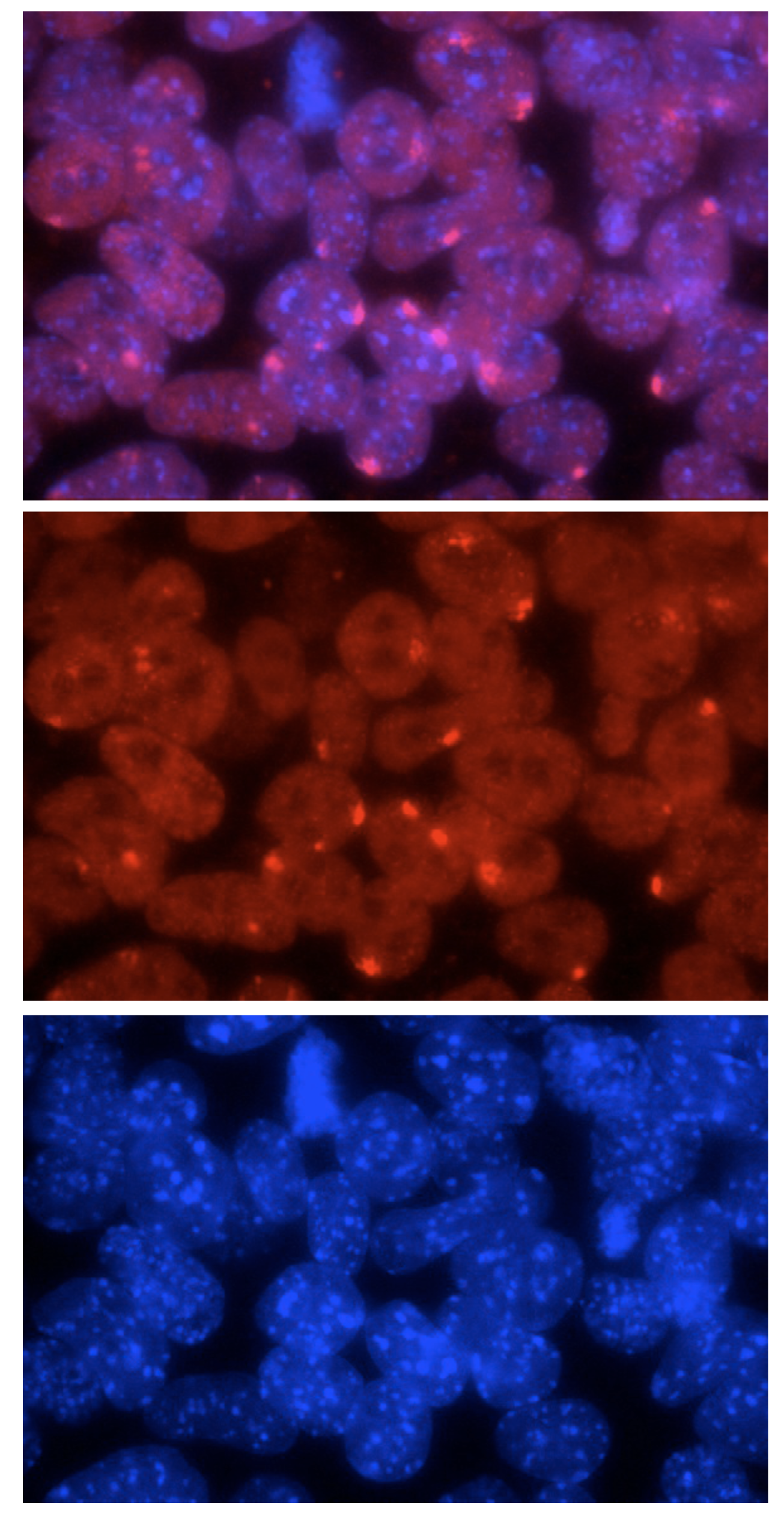

Supplement: S4 Fig — Immunofluorescence detection of Ezh2 using HP3-10 ES cells differentiated for 50 hours under the epiLCs protocol. Bottom panel: DAPI staining. Middle panel: Ezh2 signal alone. Top panel: Ezh2 and DAPI surimposed. Cells with a single nuclear foci of Ezh2 as well as cells with two nuclear foci of Ezh2 are present. (TIF) [file pone.0116109.s004.tif]
